# Implementing Whole Genome Sequencing (WGS) in Clinical Practice: Advantages, Challenges, and Future Perspectives

**DOI:** 10.3390/cells13060504

**Published:** 2024-03-13

**Authors:** Petar Brlek, Luka Bulić, Matea Bračić, Petar Projić, Vedrana Škaro, Nidhi Shah, Parth Shah, Dragan Primorac

**Affiliations:** 1 St. Catherine Specialty Hospital, 10000 Zagreb, Croatia; petar.brlek@svkatarina.hr (P.B.);; 2 International Center for Applied Biological Research, 10000 Zagreb, Croatia; 3 School of Medicine, Josip Juraj Strossmayer University of Osijek, 31000 Osijek, Croatia; 4 Greyledge Europe Ltd., 10000 Zagreb, Croatia; 5 Dartmouth Hitchcock Medical Center, Lebannon, NH 03766, USA; 6 Medical School, University of Split, 21000 Split, Croatia; 7 Eberly College of Science, The Pennsylvania State University, State College, PA 16802, USA; 8 The Henry C. Lee College of Criminal Justice and Forensic Sciences, University of New Haven, West Haven, CT 06516, USA; 9 REGIOMED Kliniken, 96450 Coburg, Germany; 10 Medical School, University of Rijeka, 51000 Rijeka, Croatia; 11 Faculty of Dental Medicine and Health, Josip Juraj Strossmayer University of Osijek, 31000 Osijek, Croatia; 12 Medical School, University of Mostar, 88000 Mostar, Bosnia and Herzegovina; 13 National Forensic Sciences University, Gujarat 382007, India

**Keywords:** whole genome sequencing, next-generation sequencing, pharmacogenomics, cancer genomics, third-generation sequencing, nanopore sequencing, variant computational analysis, multi-omics integration

## Abstract

The integration of whole genome sequencing (WGS) into all aspects of modern medicine represents the next step in the evolution of healthcare. Using this technology, scientists and physicians can observe the entire human genome comprehensively, generating a plethora of new sequencing data. Modern computational analysis entails advanced algorithms for variant detection, as well as complex models for classification. Data science and machine learning play a crucial role in the processing and interpretation of results, using enormous databases and statistics to discover new and support current genotype–phenotype correlations. In clinical practice, this technology has greatly enabled the development of personalized medicine, approaching each patient individually and in accordance with their genetic and biochemical profile. The most propulsive areas include rare disease genomics, oncogenomics, pharmacogenomics, neonatal screening, and infectious disease genomics. Another crucial application of WGS lies in the field of multi-omics, working towards the complete integration of human biomolecular data. Further technological development of sequencing technologies has led to the birth of third and fourth-generation sequencing, which include long-read sequencing, single-cell genomics, and nanopore sequencing. These technologies, alongside their continued implementation into medical research and practice, show great promise for the future of the field of medicine.

## 1. Introduction to Whole Genome Sequencing (WGS)

### 1.1. History and Evolution of WGS Technologies

Since its inception, genome sequencing has improved dramatically when it comes to cost, time, and accuracy, mainly due to the rapid advancement of technology. In just seventy years, we went from learning about the structure of DNA to sequencing the entirety of the human genome and using these data for various important purposes [1].

It all began in the 1970s and 1980s when the first attempts at DNA sequencing were made, primarily through Sanger sequencing [2]. This pioneering method relied on chain termination and electrophoresis, paving the way for the first sequencing of small genomes. However, it was a slow and financially demanding process. The 1980s witnessed the development of automatic DNA sequencing, with various techniques such as PCR sequencing and chain termination sequencing emerging. This dramatically sped up the sequencing process and significantly reduced costs. A monumental milestone arrived in the year 2000 with the completion of the Human Genome Project. This marked the first complete sequences of the human genome, revolutionizing our understanding of genes and non-coding regions. However, the real explosion of progress occurred in the 2000s with the advent of next-generation sequencing (NGS) technologies [3]. This included pyrosequencing, Illumina sequencing, and SOLiD sequencing, enabling faster and more affordable sequencing of larger genomes, including the human genome. Progress continued through the 2010s, when NGS techniques were refined and new platforms like Oxford Nanopore and PacBio technologies allowed for long-read sequencing and the unraveling of complex genome segments. Today, WGS technologies have become indispensable tools in clinical medicine and scientific research. They enable more precise diagnoses of genetic diseases, personalized medicine, and a deeper understanding of the genetic factors influencing health. The aforementioned innovations make DNA sequencing an integral part of our ability to delve deeper into genome secrets and apply them in practice.

An essential application of WGS is the discovery of genetic variants in the human genome and their association with enigmatic or well-known clinical entities [4]. By performing this early on, preventative measures can be taken to mitigate the impact of the disease. WGS provides a valuable tool in the physicians’ arsenal and produces an unprecedented amount of information that tremendously facilitates the diagnostic process. Third-generation sequencing now stands at the forefront of genome sequencing and stands to give more accurate and cost-effective results. WGS can be applied to newborn screening, cancer detection, genetic diseases, and personalized medicine [5]. It has the ability to revolutionize the way certain diseases can be diagnosed, resulting in the avoidance of long and expensive traditional diagnostic methods. Although there are advantages to this technique, the disadvantages must also be taken into account. One such disadvantage is our limited understanding of the significance of certain variants that WGS discovers. This presents a problem when trying to interpret WGS findings and determine if the discovered variant is responsible for the clinical presentation. This interpretation is further complicated by the fact that some diseases are a combined product of multiple variants, not just any single one. Excellent tools for genetic interpretation are widely accessible databases and classification algorithms that can provide physicians with supplementary data.

**Figure 1:**
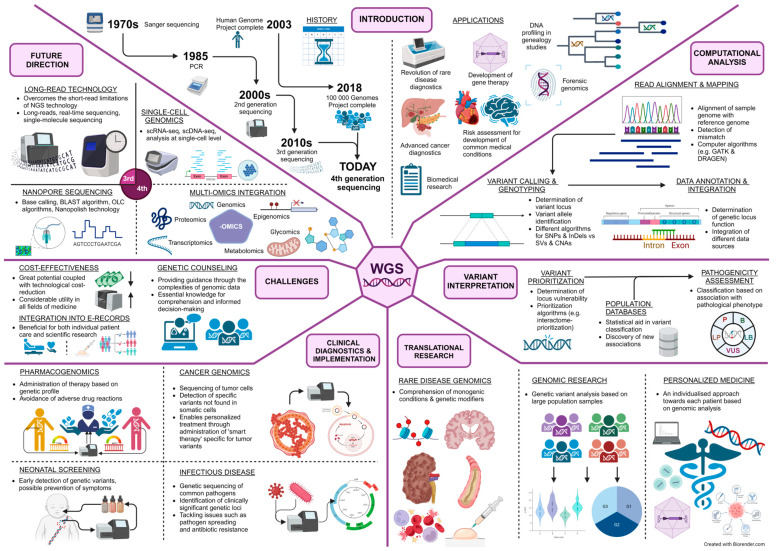
A comprehensive overview of the main aspects of WGS (created with Biorender.com). SNP—single nucleotide polymorphism; InDel—insertion and deletion; SV—structural variant; CNV—copy number variant; P—pathogenic variant; LP—likely pathogenic variant; VUS—variant of uncertain significance; LB—likely benign variant; B—benign variant, BLAST—Basic Local Alignment Search Tool; OLC—Overlap Layout Consensus.

Overall, WGS offers a massive benefit to the field of medicine. As technology progresses, the number of diseases that WGS can detect will steadily increase, as well as its accuracy. On the other hand, scientists are continuously working towards a better understanding of the data this technology provides us, resulting in increasingly accurate interpretations of results. The aim of this review was to comprehensively and clearly cover the advantages, challenges, and future perspectives of WGS in everyday clinical practice. Figure 1 depicts all the main topics covered in this review.

### 1.2. Applications of WGS in Biomedical Research

WGS has become an emerging technology as rapid strides have been made over the past few decades. WGS has revealed a wealth of information, including gene number and density, repeat sequences, non-protein coding RNA genes, and evolutionary conserved sequences [6].

WGS can detect single nucleotide polymorphisms (SNPs) in both introns and exons, which is crucial since SNPs can be attributed to a wide range of conditions [4]. In healthcare, disease susceptibility, drug responses, and physical traits can, in certain instances, be attributed to SNPs. WGS is excellent for sequencing non-coding RNA, which includes, but is not limited to, transfer RNA, ribosomal RNA, small nuclear RNA, and miRNA [7]. miRNA is a key area of study because it has an important regulatory function, whereas SNPs can cause an increase in oncogenic risk.

Although there are many more SNPs yet to be discovered, the technology is still relatively new, and time is bound to answer questions that scientists are asking today. WGS has the ability to revolutionize the way preventative medicine is conceptualized. Through WGS, physicians will have the ability to determine individual genetic profiles, allowing for prediction of likelihood of future disease manifestation with considerable accuracy [5].

WGS is slowly becoming more and more economically feasible, opening the opportunity for great benefits [8]. For example, it can detect genetic variants that can cause rare immunological disorders. WGS has the potential to dramatically reduce the time spent on the diagnostic odyssey as well as overcoming the large costs associated with missed or delayed diagnosis. WGS essentially circumvents this costly process by a one-time, relatively inexpensive test to reveal a vast amount of information that traditional methods cannot. This allows for real actionable steps to be taken to mitigate or altogether prevent certain diseases.

Although the technology is efficient, some of the data gathered from WGS are hard to translate into actionable measures [9]. There is a significant increase in the number of variants of uncertain significance. Scientists are, however, breaking through this barrier and learning to make the connections between variants and phenotypes. For example, variants of uncertain significance (VUS) are stored in a database, which allows different laboratories to collaborate and better understand which role they play in the disease [10]. Consequently, the rate of diagnosis will likely steadily increase in the future as the mysteries of the genome begin to unravel.

### 1.3. Revolutionizing Rare Disease Diagnosis with WGS

One of the key advantages of WGS compared to whole exome sequencing (WES) is the ability to analyze non-coding regions of the genome. Non-coding DNA contains various components, including repetitive sequences (telomeres, centromeres, satellite DNA), sequences encoding different types of non-coding RNA molecules, and numerous regulatory elements (promoters, enhancers, and silencers). Non-coding RNA molecules and other regulatory elements play a crucial role in gene expression control. These genomic loci are particularly important in diagnosing multifactorial genetic diseases. WGS allows for a detailed analysis of non-coding regions, providing the opportunity to identify variants that can affect gene regulation and consequently disease development [11,12,13].

Uncommon medical conditions, collectively known as rare diseases, encompass a vast array of over 8000 unique disorders, most of which stem from genetic origins. While each of these conditions is individually infrequent, their combined impact affects a considerable segment of the population, with a prevalence ranging from 6% to 8%. A study conducted as part of the 100,000 Genomes Project unveiled that WGS played a pivotal role in providing diagnoses for 25% of participants grappling with rare disorders [14]. This innovative approach demonstrated its ability to detect conditions that might have otherwise eluded traditional diagnostic methods. Furthermore, a more recent investigation shed light on the potential of tailoring WGS analyses to individual patients, a practice that could significantly augment the diagnostic rates of these conditions [15]. The acceleration in diagnosis is particularly valuable for certain rare diseases, such as primary mitochondrial disease phenotypes—a cluster of inherited disorders arising from mutations in either mitochondrial or nuclear DNA.

The non-coding regions of the genome, which make up the vast majority of our DNA (98.5%), were long considered “genomic junk” because they did not code for proteins. However, with the completion of the Human Genome Project (HGP) and advancements in next-generation sequencing (NGS) technology, research has increasingly suggested that non-coding regions of the genome play a pivotal role in gene regulation and can have a significant impact on disease onset [16]. WGS in particular offers the opportunity to uncover variants in non-coding regions, opening new perspectives in understanding the origins of genetic diseases in children [13]. Comprehensive genomic analysis can reveal the causes of rare inherited diseases, including mitochondrial disorders, neurological conditions, metabolic disorders, hematological disorders, and bone and soft tissue development disorders, as well as assess the risk of multifactorial diseases like diabetes and childhood obesity [13,17]. Given the continuous advancement of scientific knowledge, WGS provides the ability to discover new genetic changes that may lead to diseases. Through secondary findings, it may also enable the prevention and timely treatment of health issues that were not the initial reason for testing [18]. Such proactive healthcare, within the context of pediatric preventive medicine, yields better treatment outcomes and ensures disease prevention before advanced disease stages requiring challenging treatments occur [18,19].

Mutations in regulatory elements within non-coding regions lead to changes in gene expression, which can significantly impact phenotypic manifestations and disease development. For instance, mutations in promotors, enhancers, or silencers can affect the binding of transcription factors and alter the expression levels of specific genes, resulting in disease development [20,21]. Detecting variants in regulatory regions through WGS provides the opportunity to identify new variants as causes of genetic diseases in children, in which previous analyses failed to establish a cause in coding DNA. Identifying specific variants in regulatory elements may enhance our understanding of the underlying molecular mechanisms. This can lead to the discovery of new therapeutic targets and the development of novel therapeutic strategies [22]. Additionally, gene variants in non-coding regions may have implications for understanding complex genetic diseases involving the interaction of multiple genes and environmental factors. For example, changes in specific genes may increase susceptibility to certain environmental factors, such as susceptibility to infections like *H. pylori*, which can result in an increased risk of multifactorial diseases such as gastric ulcers and stomach cancer [23,24].

Clinical genome analysis can be divided into three phases: primary, secondary, and tertiary analysis. Primary analysis encompasses the technical components of next-generation sequencing, including DNA extraction, library preparation, and preliminary sample quality control. Secondary analysis involves bioinformatic data processing from sequencing, including aligning the obtained sequence with the reference human genome and additional computational operations to correct potential analysis errors [4]. Finally, tertiary analysis involves variant interpretation, including variant annotation, filtering, clinical classification, result interpretation, and the generation of a medical report for genetic testing. This review will cover primary, secondary, and tertiary analysis, with a specific focus on clinical interpretation and the application of WGS in everyday clinical practice.

## 2. Computational Analysis of WGS Data

### 2.1. Alignment and Mapping of Sequencing Reads

In WGS, alignment and mapping of sequencing reads implies arranging the reads so that values at specific points can be compared [25]. At these points, we expect these values to be equal, as these are homologous points in the reference genome, and interpret any mismatch as a variation in the sequence being tested. In clinical practice, this is the first step in genetic data analysis and involves aligning the sample genome to a reference genome and observing potential differences, which are later interpreted as genetic variants. Approximating homology between two sequences using similarities in sequencing reads was pioneered by Needleman and Wunsch in the form of optimal pairwise global alignment. This then led to the development of optimal pairwise local alignment by Smith and Waterman, which was designed for the alignment of subsequences. The conceptual solution for effective whole genome alignment was to make a division into subsequences and then apply local alignment algorithms [26].

In clinical genetic sequencing today, analysis of sequencing reads is performed in processes referred to as data analysis pipelines. These can be categorized into upstream pipelines, which carry out the task of read alignment and mapping, and downstream pipelines, designated for genetic variant calling. A study by Betschart RO et al. compared two alignment and mapping approaches in WGS, GATK utilizing BWA-MEM2 2.2.1, which is most frequently used, and DRAGEN 3.8.4. While the authors conclude that DRAGEN is superior to GATK, they also highlight the important aspects of comparison when it comes to systems for genome alignment and mapping. Firstly, the comparison of alignment systems was broken down by single nucleotide variants (SNVs) and insertion-deletion variants (Indels). SNVs represent value changes at homologous points in compared reads and will be detected as a mismatch. Indels, on the other hand, represent an added or missing value, which causes the entire read to shift. It is due to this difference that SNVs and Indels represent different challenges for alignment systems. Furthermore, comparison was categorized by Indel size, which can imply the gain or loss of multiple values in the sequence, as well as whether a coding or non-coding region is in question. Once the algorithms have been stratified in accordance with these differences, parameters such as time to completion and precision in detection could be observed [27].

Genetic mapping has become a great asset in the personalized medical approach in many medical disciplines, but perhaps most evidently in oncology, with ongoing projects which aim to complete the global mapping of the cancer genome. Ganini C. et al. extensively highlight this matter in their comprehensive paper, discussing all aspects of this line of research in modern times. Neurology is another of many disciplines utilizing genetic mapping, as is highlighted in research by Png G. et al. which describes the mapping of the serum proteome to neurological disorders [28,29].

### 2.2. Variant Calling and Genotyping

Variant calling is the process of identifying genetic variants from received sequencing data [30]. This is the next step in data analysis following alignment and is performed by downstream data analysis pipelines. Variant calling can be categorized into germline and somatic variant calling. Germline variant calling implies that the interpretated variants are generally in a similar haplotype configuration of the reference genome while respecting the paradigms of mendelian principles in most cases. However, somatic variant calling allows for the existence of multiple cell lines and the development of frequent de novo mutations violating Mendelian principles. Somatic variant calling is useful in detecting cell mosaicism within an individual and has an especially important application in genotyping tumor cells.

Variant calling algorithms can also be categorized by different types of genetic variants. SNVs and smaller Indels, up to 20 base pairs, can be detected directly after alignment and often require only minor local realignment once a candidate site has been detected. On the other hand, structural variants (SVs) and copy number variations (CNVs) are not as simple to precisely determine. These algorithms primarily rely on the depth of coverage, as well as assembly-based sequence reconstruction after a candidate for SV or CNV has been identified [31].

A study published by Pei S. et al. systematically evaluated different variant callers on 12 next-generation sequencing datasets for both germline and somatic variants. The germline callers Sentieon, GATK, and DeepVariant all had an F1 score of over 0.99 and a 30x coverage, results which show a high sensitivity and accuracy in all three systems in analyzing germline variants. Somatic callers such as Mutect2 and TNscope were tested in calling somatic variants. The systems achieved high F1 scores overall, but more interestingly, a correlation between tumor sample purity and accuracy was noted. Both systems showed better accuracy in calling both SNVs and Indels as the tumor sample purity increased. Overall, the authors concluded that careful selection of variant caller, depending on the circumstances, is of great importance to reliable variant detection [32].

Variant genotyping entails a different process than variant calling. Calling merely provides evidence of a genetic variant in a specific gene locus. Variant genotyping is the process of identifying the specific allele that was detected by calling and is therefore the next step in genetic data analysis. Determining the specific change that has occurred has great value, as variants are later classified to determine their clinical significance. Similar to variant calling, SV genotyping is much more complex than the genotyping of SNVs or Indels, as is highlighted in a comprehensive evaluation by Duan X. et al. [33].

### 2.3. Structural Variant Detection and Analysis

The term structural variant refers to a larger genetic alteration and encompasses several types of variants such as deletions, insertions, duplications, translocations, and inversions. These are categorized differently from Indels, as they are at least 50 base pairs in size. It is no surprise that such variants pose a challenge when it comes to computational data analysis. The general steps of structural variant detection and analysis are the same as with SNVs and Indels, and they involve alignment, calling, and genotyping [34]. However, the algorithms used for SV analysis are specifically designed for this purpose. SV discovery and genotyping is of grave importance in clinical genetics, as it has been shown that these variants can have important roles in phenotype diversity, as well as complex genetic conditions [34].

In the evaluation published by Duan X. et al., five long-read systems for SV genotyping were evaluated, including cuteSV, LRcaller, Sniffles, SVJedi, and VaPoR. LRcaller and cuteSV had the best F1 scores for insertions and deletions, while LRcaller gave the best performance with duplications, inversions, and translocations. Firstly, the authors noted that the accuracy of the algorithms is inversely proportional to the size of the SV. This would indicate that larger SVs pose a greater challenge to analyze, which is concurrent with the difference in both size and analysis complexity between SVs and Indels. Secondly, the authors concluded that the algorithm accuracy is greater in the case of insertions and deletions than in duplications, inversions, and translocations. One of the possible reasons for this is that complex genetic alterations such as translocations and inversions can often be accompanied by additional changes such as deletions or duplications at the site of separation or joining of genetic material. Finally, the conclusion regarding depth of coverage was that analysis at a depth of coverage of 20× produces diminishing returns in the F1 scores [33].

As highlighted in a recent review by Romagnoli S. et al., Oxford Nanopore Technology developed the first sequencing system that uses nanopores as biosensors to sequence longer DNA molecules. The authors concluded that this novel system could resolve the problem regarding the sequencing of complex SVs. As for clinical applications, the authors discuss prenatal diagnostics, as well as cancer profiling [34].

### 2.4. Data Integration and Annotation in WGS

While the so-far-covered process of DNA analysis provides the exact sequence of base pair values and their potential alterations, it gives little information about its functional regions. Genomic annotation is the process of determining which elements of the DNA sequence hold which function [35]. The most frequent example is a protein-encoding gene, but others include different regulatory DNA regions. Annotation gives meaning to the analyzed sequence and provides necessary information for clinical evaluation of sequencing.

The process of annotation has evolved substantially over the last three decades, and the techniques used can be categorized into several major stages. The beginning of genomic annotation was marked by computer algorithms in the 1990s, which were designed to predict protein-encoding regions. From that point on, the focus was primarily on the annotation of species-specific reference genomes constructed by statistical methods. In the last few years, however, as multi-omics became a staple of innovative medicine, annotation of other functional DNA units, such as regulatory elements, has become common, if not a standard [36].

The process of data integration entails combining results produced by different sources into a single, uniform view or format. Wen B. et al. designed and proposed an efficient integration algorithm, which the authors called the NGS-Integrator. Their published paper highlights the aspects of data integration, as the algorithm allows for the integration of multiple datasets generated by the same method but also datasets generated by different methods. The result of this process is one single track produced by reformatting multiple genome-wide sequencing results. The authors conclude that a time and memory-efficient algorithm can significantly facilitate downstream analysis such as identifying regulatory DNA domains. In genetic research and practice, the process of data integration is essential for the reproducibility of the analytic process, as well as the comparison of experimental results [37].

## 3. Interpreting Genetic Variants in WGS

The American College for Medical Genetics (ACMG) classification classifies variants into five categories: pathogenic (P), likely pathogenic (LP), variant of uncertain significance (VUS), likely benign (LB), and benign (B). The classification criteria will be further described in the following text.

### 3.1. Functional Annotation and Prioritization of Variants

Functional annotation and prioritization of genetic variants is an essential step when it comes to estimating the significance of a genetic variant concerning a certain clinical phenotype [38]. When using WGS for diagnostics of rare diseases, determining which of the many discovered variants are responsible for the presented disorder can be a great challenge. One aspect of variant prioritization is determining the mutation tolerance of the specific gene locus in question. As several studies have shown, mutation rates vary across the human genome, meaning some loci are more vulnerable to mutations than others. An example of this was demonstrated in a study by Petrovski S. et al., in which the authors concluded that gene loci responsible for Mendelian genetic diseases are significantly more susceptible to variation occurrence [39]. Another aspect of variant prioritization is determining the mutational architecture of the variant and its correlation to a given phenotype. This is an important aspect, as it is well-known that abnormalities in different regions of the same gene can lead to different clinical manifestations. Finally, the process of variant prioritization involves determining the mode of inheritance, zygosity, and origin of a variant. This is essential when observing a patient and his condition relative to his family members, who might also be candidates for WGS. As an example, a heterozygous variant found in a patient, but also other unaffected members of his family, can often with great probability be ruled out as the cause of an autosomal dominant condition with full penetrance. Likewise, if the same pathological patterns repeat within a family intergenerationally, WGS testing of multiple family members can quickly elucidate which variant might be responsible [38,39].

The modern process of variant prioritization utilizes highly effective prioritization algorithms. One such example can be found in a study published by Schluter A. et al., in which the authors tackled the problem of diagnosing genetic white matter disorders (GWMDs) [40]. The authors derived a seed group of GWMD-related genes from their patients’ Human Phenotype Ontology terms. Following this step, an interactome-prioritization algorithm was applied, based on network expansion of the created seed group. The term interactome refers to all molecular interactions within a particular cell. The described algorithm observes the molecular interactions between products of genes from the seed group and other molecular products that have their own corresponding genes. These genes then become the next candidates for testing, and observing all interactions of their products grows the network even further. Using this algorithm, the authors were able to discover novel candidate genes for GWMDs and deemed their method more time-efficient than the classical targeted diagnostic approach.

### 3.2. Variant Databases and Population Frequency Analysis

Genetic variant databases are an important tool in the interpretation of genetic variants, as well as the discovery of new relationships between genes and diseases. Over the last decade, several projects, pioneered by the 1000 Genomes Project, have undertaken the task of generating and aggregating large collections of human genetic sequencing data [41]. As a result, comprehensive and accurate genome-wide estimations of variant frequencies in the human population have become publicly available. These large-scale variant databases are not without their limitations, with the most obvious being extremely difficult quality control. The data curated in these databases are acquired from an immensely large number of different sources, from large-scale population studies to individual reports made by clinicians. Major examples of such databases are GnomAD, OMIM, HGMD, Uniprot, dbSNP, PubMed, ExAC, and ClinVar, which are responsible for the curation of a large number of reported variants and their frequency analysis. Additionally, web-based tools like the UCSC Genome Browser and Ensembl facilitate the visualization and analysis of genomic data and contribute to the curation of reported variants. Determining the frequency of a specific genetic variant can be a useful step in its interpretation. While the low occurrence of a variant is not sufficient to declare it pathogenic, there is an undeniable correlation between the rarity and pathogenicity of genetic variants [42].

Apart from its usefulness in individual phenotype assessment, population frequency analysis also plays a vital role in genetic epidemiology. On one hand, it can be used to determine the frequencies of variants for autosomal recessive disorders within a subpopulation or nation. The findings of such studies can prove immensely significant, as they can draw attention to abnormally high occurrences of rare conditions in a specific region and lead to the implementation of new health protocols, such as genetic screening. One example is the study published by Scotet V. et al., in which the authors discuss the epidemiology of cystic fibrosis and genetic-based health policies, one of which is genetic screening [43].

On the other hand, population frequency analysis is also of great significance in cancer epidemiology. An example is a research paper published by Zavala V.A. et al., which offers a comprehensive view of the genetic epidemiology of breast cancer in Latin America. The authors evaluate the available knowledge of breast cancer epidemiology, as well as genome-wide association studies perfomed in countries in Latin America. In their conclusion, a population-specific frequency analysis is prudent in constructing the correct risk prediction model, as a model constructed on European population data can prove inaccurate in this case [44].

### 3.3. Clinical Significance and Pathogenicity Assessment

Determining the pathogenicity and clinical significance of a genetic variant represents the final step in individual WGS testing. While this is the most important aspect of clinical genetics, it can also be the most challenging due to the complexity of variant classification. Early variant classifications categorized genetic variants into two groups. Variants with a population frequency higher than 1% were labeled as polymorphisms, while variants with frequencies lower than 1% were called mutations [45]. This, however, often led to confusion, as this classification provided no information on the respective variant’s impact on a clinical phenotype. In 2015, ACMG proposed a new classification system that categorized variants by likelihood of phenotype impact or pathogenicity. Pathogenicity, however, is always interpreted in the context of a specific condition, as well as the mode of inheritance [46]. Additionally, models exist which utilize a Bayesian framework, as well as VCEP protocols [47].

As previously stated, genetic variant databases and population frequency analysis have a vital role in the classification of genetic variants. For this reason, each variant classification is accompanied by a category showing its corresponding level of evidence. These levels of evidence are (1) population, (2) computational, (3) functional, and (4) segregation data. A stronger level of evidence for a certain variant classification implies a larger sample on which the variant has been observed. On the other hand, underreported variants often fall into the VUS category and are reclassified as the level of evidence increases [42].

Modern technology has given way to computational, or in silico, prediction of variant pathogenicity. Garcia F.A.O. et al. provide an overview of in silico prediction tools from the early 2000s to today. From the mid-2000s, in silico prediction tools examined the conservation of DNA regions in order to assess the likelihood of a variant having an impact on a clinical presentation. Once large-scale databases of sequencing data emerged, the capabilities of these tools improved, as they had a much larger sample to derive data from. Machine learning systems (MLSs) are also a noteworthy asset in variant analysis [48]. Supervised MLSs require large databases in order to be “trained” to assess pathogenicity but can utilize a number of biochemical and mathematical parameters which are out of reach for tools focused on conservation. On the other hand, unsupervised MLSs undergo no training process and are therefore considered less reliable but also less biased, as their analytic process is not dependent on the sample they are “trained” with. The authors conclude that in silico prediction tools have an important role in providing evidence for variant classification, and their further development will provide better diagnostic accuracy in clinical genetics [49].

### 3.4. Interpretation of Non-Coding Variants

Coding genetic regions make up only 1% of the human genome, while the rest pertains to non-coding regions [50]. The drawback of whole exome sequencing (WES) and many classifying algorithms as diagnostic tools is their exclusivity towards coding variants. Generally, variants in non-coding regions include deep intron variants, promotor or enhancer variants, structural variants, and chromatin configuration variants. Despite not coding for specific proteins, variants in these regions can still affect their function and are associated with medical conditions.

The promise of WGS is a complete overview of the human genome, coding and non-coding regions alike, making it a far more powerful tool for data collection and diagnostics [51]. With the integration of data science and data analytics in modern medicine, WGS will provide a much greater volume of data, likely sufficient for the optimization of machine learning and deep learning models. This might, in turn, facilitate the development of new classification algorithms with a much broader capacity for pathogenicity determination, including non-coding variants. Examples of in silico studies on non-coding variants can already be found in the literature, conducted on large data repositories for non-coding regions such as HaploREG and RegulomeDB [52].

## 4. WGS in Translational Research

### 4.1. Mendelian Disorders and Rare Disease Genomics

Rare Mendelian diseases, disorders caused by a single gene, show considerable variation in clinical appearance and severity, conveying the principle that many other factors affect the outcome of the disease. Genetic modifiers are genetic loci that may affect how disease-causing mutations manifest themselves [53]. They play a critical role in regulating the phenotype of Mendelian diseases, as they may either lighten or aggravate the symptoms associated with the disease. Monogenic disorders, commonly referred to as Mendelian disorders, are a class of hereditary diseases brought on by changes in a single gene [54]. Mendel’s rules of inheritance apply to many conditions which show recognizable inheritance patterns such as autosomal dominant, autosomal recessive, or X-linked inheritance. Huntington’s disease, sickle cell anemia, and cystic fibrosis are a few examples of Mendelian illnesses. It is worth noting that these diseases can have a complex genetic etiology. For example, 16 different genes have been associated with an osteogenesis imperfecta phenotype [55]. These complexities are relevant, as they can lead to multiple potential genetic therapeutic approaches [56,57].

Genetic modifiers affect the way a disease presents itself through many different mechanisms including gene expression, protein function, and cellular pathways [53]. Identifying and labeling genetic modifiers in rare Mendelian diseases can be a difficult task. It is difficult to acquire sufficient data for analysis because of the rarity of these diseases and the complexity of the genetic landscape. However, by employing certain experimental research approaches such as genome-wide association studies, whole exome sequencing, and functional studies in model organisms, studying said genetic modifiers can be made easier.

The clinical ramifications of comprehending genetic modifiers are critical. It is now possible to predict illness outcomes more accurately, classify individuals into various risk groups, and create individualized treatment plans by identifying specific modifiers. Furthermore, the potential for designing pharmaceuticals that specifically target or manipulate the pathways that these modifiers affect is also feasible. Genetic modifiers play a significant role in the development of the clinical presentation and severity of rare Mendelian diseases. Understanding these modifiers opens up possibilities for better diagnosis, prognosis, and therapeutic approaches, ultimately improving patient care in the setting of rare genetic disorders [58].

### 4.2. Genomic Medicine and Precision Healthcare

Recently, there have been many advances in genetics that hold the potential to revolutionize healthcare. Genomic medicine, precision medicine, and personalized medicine are all important interrelated practices that are prevalent in clinical practice [59]. Genomic medicine refers to the application of a patient’s genomic data, such as DNA sequence variants and other genetic traits, to influence clinical judgment. In order to improve diagnostics, predict illness risk, and create targeted therapeutics, genomic medicine strives to understand the genetic basis of diseases. An excellent example of this approach is the prediction of illness risk in cardiovascular diseases [60].

Precision medicine is an approach that bases treatment choices on an individual’s unique genetic makeup, environmental influences, and lifestyle choices. It entails customizing medical interventions to each patient’s unique traits in an effort to maximize therapeutic results and reduce side effects. Although it utilizes genomic data, precision medicine also accounts for non-genetic factors.

Precision medicine is seen as a type of clinical practice within personalized medicine, which covers factors other than genetics [61]. In addition to genetic and clinical data, it considers the preferences, values, and circumstances specific to each patient. Personalized medicine emphasizes the significance of adapting medical choices to the particular requirements and traits of each patient. The fields of personalized, precision, and genomic medicine are linked and have similar aims. Rather than being mutually exclusive, these phrases indicate various viewpoints within the developing field of personalized healthcare. However, there are still obstacles in the way of implementing genomic medicine, precision medicine, and personalized medicine [62]. These issues include the necessity for interdisciplinary cooperation, complex genetic data interpretation and communication, integration into current healthcare systems, and ethical issues. These concepts are fluid and are constantly evolving, so developments in technology, data analysis, and knowledge of the genome will continue to shape the field. To fully achieve the potential of genomic medicine, precision medicine, and personalized care, it is essential to continue research, education, and collaboration with other researchers with vested interests.

## 5. WGS Applications in Clinical Diagnostics

### 5.1. Prenatal and Neonatal Genetic Testing

Newborn screening (NBS) has become an essential tool for disease prevention and treatment from an early age. It has taken a proactive approach rather than a reactive approach, allowing for disorders to be discovered in their earlier stages [63]. With the advent of next-generation sequencing and its application in newborn screening, two advantages present themselves. WGS can predict many more diseases while simultaneously improving the accuracy of results, essentially serving as a preventative measure in neonatal and pediatric care [64]. One benefit that WGS provides is that it completely circumvents the arduous and costly process of traditional diagnosis.

Furthermore, due to the extensive information that WGS provides, physicians can predict with a greater degree of accuracy which diseases patients can develop and what the probability of such a development is [65]. With WGS, predicting disorders prior to symptom onset, ten, fifteen, or even twenty years in the future might be possible. Given this information, immediate steps can be taken for early monitoring and treatment, which mitigates the disease’s emotional, physical, and financial impact on both the afflicted as well as their family members. WGS data can also be used for genetic counseling for potential future pregnancies [66].

With the wealth of information that WGS provides, clinicians would be able to screen for both metabolic and non-metabolic disorder genetics [63]. With the advantages presented, WGS in NBS can greatly expedite the process of diagnosis and treatment and can serve as a vital tool for both physician and patient.

### 5.2. Cancer Genomics and Precision Oncology

WGS has the ability to detect important somatic mutations in tumor tissue [67]. Through early detection of cancer mutations, each malignant disorder can be characterized in great detail, which facilitates a personalized approach.

Several factors including different input amounts, tumor purity, various library construction protocols, sequencing instruments, and bioinformatics pipelines can impact somatic mutation detection. WGS generated better data than whole exome sequencing (WES), which had higher G/C content and more adapter contamination [68]. Furthermore, formalin-fixed paraffin-embedded (FFPE) blocks showed more DNA degradation in WES as compared to WGS, and as a result, WGS is better suited for this method of tissue preservation. Mutation callers such as MuTect2 or Strelka2 can be used [69]. Strelka2 overall had the best reproducibility for WGS but the worst in WES runs, while MuTect did consistently well in WES. WGS sequencing has much more reproducibility and consistency than WES and is subject to less variation.

The importance of precision oncology is not only highlighted by examples with somatic variants but germline variants as well. One excellent example of the importance of WGS in cancer treatment can be found in hereditary gynecological cancers, such as ovarian cancer and breast cancer [20]. The genetic etiology of these conditions is most often associated with germline variants in the *BRCA1* and *BRCA2* genes, as well as *BARD1*, *PALB2*, *ATM*, *MLH1*, *MSH2*, *AKT1*, *CDH1*, *CTNNB1*, *MSH6*, *NBN*, *PIK3CA*, *PMS2*, *PRKN*, *STK11*, *TP53,* and others. Understanding the underlying genetic mechanisms of these cancers has led to the development and application of novel therapeutic agents, such as PARP inhibitors. It has been shown that *BRCA1/2*, alongside other genes, take part in the repair of double-strand DNA breaks by inducing homologous recombination. As this mechanism is defective, tumor cells greatly rely on the PARP repair mechanism, unlike healthy cells with functional homologous recombination. For this reason, PARP inhibitors selectively cause DNA damage accumulation in tumor cells, leading to their apoptosis [70].

A recent publication greatly emphasizes the importance of cancer genetics. Using WGS technology, the authors analyzed 13,880 solid tumor genotypes. The results of the study provided a great insight into the statistics of cancer genomics, likely greatly facilitating further research in the field of oncology [71].

### 5.3. Pharmacogenomics and Personalized Medicine

With the significant decrease in price for DNA sequencing, a new field known as pharmacogenomics (PGx) is being pioneered by scientists. PGx is the study of how genetic factors impact the way drugs are metabolized in an individual organism [72]. Through genome sequencing, PGx will be able to boost therapeutic benefits and reduce negative side effects. It has been theorized that genetic factors can account for up to 95% of an individual’s drug response, and their contribution to the total number of adverse reactions is estimated to be as high as 20% [73]. Genome sequencing reveals an enormous amount of information and enables proper drug and dose selection through PGx [74].

There are several examples of PGx proving very useful in clinical practices. Abacavir is frequently used in combination with other antiretroviral drugs to treat HIV. However, between 5 and 8% of infected individuals can develop a very severe hypersensitivity reaction due to a major histocompatibility complex I allele (HLA-B*5701) [75]. Through PGx screening of this allele, hypersensitivity towards Abacavir decreased by 60%. The results from another study found that the presence of the allele is correlated with Abacavir sensitivity, thus illustrating the importance of PGx testing when prescribing medication. Another example drug is codeine, which has demonstrated variable toxicity dependent on CYP2D6 variants [76]. In the same manner, statin efficiency and toxicity have shown variability with different CYP3A4 and SLCO1B1 variants. Up to 10% of patients exhibit muscular symptoms, which might be avoided with a personalized PGx approach [77]. Studies have also shown that clopidogrel has variable efficiency in different CYP2C19 genotypes [78]. Additionally, PGx testing can be beneficial when prescribing warfarin, as well as novel oral anticoagulant therapeutics, as it allows for the identification of clinically relevant polymorphisms [79,80].

Studies conducted by the University of Chicago and St. Jude Children’s Hospital both claimed that PGx was important and feasible [81,82]. In another study by the Mayo Clinic, the authors claimed that between 91 and 99% of the population had one PGx variant that could cause an adverse reaction to drugs [83]. For example, variation in the CYP2D6 gene, which is responsible for drug PGx, can have vastly different results, from negligible effects to cases of overdose. PGx addresses this issue by sequencing a person’s genome and then recommending whether to take certain medications. The progress towards PGx is continuously steady, as tests are being conducted in approved laboratories and are even now starting to become mandatory in certain countries. Baylor Institute of Medicine includes PGx for both warfarin sensitivity and clopidogrel metabolism, enabling patients to take the medication best suited for them. Additionally, PGx has a big role to play in moderating drug administration in psychiatry and has already proven useful in certain clinical cases [84].

PGx has the ability to revolutionize the way healthcare is administered and could predict with a great deal of certainty which treatment option is the most appropriate [85]. Drug side-effects can be a great treatment obstacle, and PGx tackles this issue by providing solutions specifically tailored to patients’ genetic code. PGx can maximize the efficacy of drugs and minimize the debilitating side effects, ensuring the best healthcare is being administered to patients [86,87].

However, an opposing viewpoint regarding the clinical utility of WGS in pharmacogenomics can also be found in the literature and is therefore worth mentioning. More skeptical authors have arrived at the conclusion that WGS does not warrant clinical implementation in this regard due to insufficient knowledge and an absence of clear guidelines. In their viewpoint, the expectation of improved clinical outcomes and better informed clinical decision-making due to PGx is still out of reach and warrants further research [88].

### 5.4. Infectious Disease Genomics and Outbreak Investigations

Outbreak investigations are nearly always employed at the start of an outbreak to determine the specific strain, method of spreading, and ways to prevent it [89]. Through this information, scientists can begin to tackle the problem methodically and use WGS of the pathogen to aid in their efforts.

Currently, antibiotic resistance has become one of the largest public health crises, with even the strongest antibiotics having little to no effects on certain bacterial strains. WGS can be used to predict resistance phenotypes in *E. coli* and *S. aureus*, which have become increasingly resistant to antibiotics [90]. Furthermore, mutations in these bacteria can be detected by WGS. Evidence from WGS has proven that pneumococcal bacteria have begun to capsule switch, preventing them from becoming phagocytized by the immune system [91]. This information allowed scientists to develop a more effective vaccine better suited to counter pneumococcal bacteria. Furthermore, by showing the entire genome and its subsequent evolution, scientists can determine what allows bacteria to become virulent as well as the cause of their resistance. They can then develop ways to combat the bacteria and create vaccines for future mutations, thus minimizing the effects of the disease.

Understanding the cause of pathogen spread is crucial in outbreak investigations by public health officials. For example, during an outbreak of MRSA in China, scientists learned through WGS that the sasX gene was crucial for the successful spread of the pathogen [92]. In addition, WGS can also be used to characterize different types of strains. After the rubella virus was eradicated in the United States, cases still appeared. After performing WGS on the genetic profile of these viruses, it was determined that they were brought from foreign entities, as the profile matched the rubella virus strains to different countries [93,94]. Similarly, hospitals that persistently suffered from *C. difficile* outbreaks managed to uncover the underlying cause of the infections using WGS [95].

WGS offers invaluable information to outbreak investigations and aids scientists in ending current outbreaks as well as providing preventative measures for future outbreaks. As WGS technology progresses, outbreak investigations can become more efficient and accurate and less costly. It offers the opportunity for scientists to enhance their understanding of resistance and allows them to create much more effective medicine in their fight against ever-mutating pathogens.

## 6. Implementation and Challenges of WGS in Clinical Practice

### 6.1. Clinical Utility and Cost-Effectiveness of WGS

WGS offers a comprehensive analysis of an individual’s entire genetic code, providing invaluable insights into their genetic makeup and potential health risks. One of the key advantages of WGS is its ability to diagnose rare and complex genetic disorders with a high degree of accuracy. This not only improves patient outcomes but also reduces the burden of prolonged and inconclusive diagnostic processes [96]. Moreover, the cost-effectiveness of WGS has improved over the years, making it a viable option for clinical use. The decreasing cost of sequencing and data analysis, coupled with the potential for early disease detection and prevention, positions WGS as a valuable investment in healthcare. In addition to diagnosing rare diseases, WGS plays a crucial role in oncology, pharmacogenomics, and personalized medicine. It allows oncologists to identify specific genetic mutations in cancer patients, guiding the selection of targeted therapies for better treatment outcomes.

Another aspect of the cost-effectiveness of WGS is the elimination of the necessity for additional diagnostic procedures. An excellent example of this is the use of whole exome sequencing in the diagnostics of autosomal genetic diseases. While WES has been a diagnostic standard for these conditions for a long time, its results can be inconclusive and appear as a diagnostic “dead-end”. A recently published study observed the utility and benefit of WGS testing in WES-negative patients [97]. The authors concluded that this was a beneficial approach, as new and useful data were obtained for a number of patients in the cohort. Based on their results, they propose the integration of WGS into the diagnostics of autosomal disorders.

WGS offers significant clinical utility and cost-effectiveness by enabling precise diagnoses, personalized treatments, and improved patient outcomes [98]. As technology continues to advance and costs decrease, the integration of WGS into clinical practice is expected to become even more widespread, revolutionizing healthcare delivery and enhancing the quality of patient care.

### 6.2. Integration of WGS into Electronic Health Records

The integration of WGS into electronic health records (EHRs) represents a significant advancement in healthcare technology [99]. This integration offers numerous benefits, from enhancing patient care to facilitating cutting-edge research. By incorporating WGS data into EHRs, physicians can better understand a patient’s genetic predispositions to various diseases, quickly search through a patient’s genomic data, accelerate the diagnostic process, and tailor treatment plans accordingly.

Furthermore, by aggregating de-identified genomic data from EHRs, physicians can conduct large-scale studies to uncover novel insights into the genetic basis of diseases [100]. This data-sharing approach fuels medical research, potentially leading to breakthroughs in the understanding and treatment of various conditions. However, challenges such as data security, privacy, and the need for interoperability standards must be addressed for successful integration [101]. Protecting patient confidentiality and ensuring seamless data exchange between different healthcare systems are paramount concerns.

Integrating WGS into electronic health records offers a promising avenue for advancing patient care and medical research. While challenges remain, the potential benefits in terms of personalized medicine and scientific discovery make this integration a compelling area of development in healthcare technology.

### 6.3. Genetic Counseling and Patient Education in WGS

Genetic counseling and patient education play pivotal roles in harnessing the power of WGS in healthcare. In an era where genetic information is increasingly accessible, it is essential to guide individuals and families in navigating the complexities of their genomic data [102]. WGS offers numerous advantages, such as early disease detection and personalized medicine. However, it also raises ethical dilemmas, privacy concerns, and psychosocial challenges. Genetic counseling and patient education are instrumental in helping individuals and families navigate this intricate landscape [103]. They equip patients with the knowledge and emotional support needed to make informed choices about genetic testing, treatment options, and family planning.

The integration of genetic counseling and patient education is paramount in realizing the full potential of WGS in the healthcare system. These essential components empower individuals to make informed decisions about their genetic information, ultimately leading to improved health outcomes and a more equitable healthcare system.

## 7. Intergenerational Evolution of Sequencing Reads

Short-read sequencing represents the initial generation of NGS technologies that followed Sanger sequencing. The length of each individual read in this method is 75–800 bp, and the reads are then massively sequenced in parallel. This is achieved by fragmentation of the DNA strand and subsequent amplification of each short fragment [104]. Amplification is performed either by emulsion PCR or bridging PCR, depending on the sequencing platform [105]. While the technology of short-read sequencing was revolutionary at its dawn, certain shortcomings became more apparent through the years. The process of DNA fragmentation and such analysis resulted in a loss of information, which made comprehensive analysis more difficult.

The introduction of long-read technologies is now transforming genomics research by allowing researchers to explore genomes at remarkable resolution. In 2011, PacBio released their PACBio RS sequencer that employs single-molecule real-time (SMRT) technology [106,107]. This machine increased average read lengths by more than ten times.

As a result of long-read sequencing methods, genome regions that were mysteries could finally be resolved, and the complex transcriptomes have the potential to be explored in great detail [108]. Some applications of long-read technologies include WGS, RNA-sequencing, and detection of epigenetic modifications.

In the context of sequencing reads, hybrid sequencing is a third option that integrates short-read and long-read sequencing. The aim is to eliminate the weaknesses of both approaches by using the strengths of the other. Short-read sequencing, due to the fragmentation of DNA, results in an information loss, which makes certain types of variants difficult, if not impossible, to detect. Long-read sequencing overcomes this issue by removing fragmentation out of the process. However, the drawback of long-read sequencing is the occurrence of errors [105,106]. A great comparison can be found in a recently published metagenomic study, in which the authors emphasize the advantages and disadvantages of these two approaches [109].

The highlighted literature presents hybrid sequencing as a superior method. It successfully overcomes the shortcomings of both short-read and long-read sequencing by combining the two methods and utilizing the strengths of each one.

## 8. Challenges and Limitations of WGS Implementation

When considering the potential of WGS in clinical practice, current challenges and limitations must be taken into consideration. One of the greatest challenges of clinical genetics is the clinical interpretation of non-coding variants. While great advances have been made in the field of in silico prediction tools for this very purpose, this still remains a formidable barrier to the full realization of WGS’s clinical utility. Building precise models based on large training databases remains a challenge due to issues such as overfitting and overgeneralizing variant effects [110]. This lack of knowledge and understanding leaves room for considerable uncertainty in the clinical diagnostic process.

Another issue is presented by the term “variant penetrance”. Pathogenic variants of low penetrance will often not lead to a pathological phenotype. In WGS testing, low-penetrance pathogenic variants can be interpreted as a “false positive” result, setting the clinician on an incorrect diagnostic course. While false positive results are arguably better than false negative results, they can still cause the patient unnecessary emotional distress, as well as lead to further medical actions, which are in that case unwarranted [111].

Difficulties with WGS diagnostics can be found in patients with non-Mendelian genetic disorders [112,113]. One such example is the paper published by Fang H et al. in 2017. The authors applied an integrated WGS-HPO pedigree to diagnose a patient with Prader-Willi syndrome. They concluded that relying solely on utilizing WGS would not have been sufficient to make the correct diagnosis in some cases, due to the complexity of the underlying genetic and epigenetic error. In cases of Prader–Willi syndrome, approximately 25% of cases are associated with uniparental disomy, and through WGS, uniparental isodisomy can be detected. The limitations of WGS testing can be overcome in certain cases by “trio testing”, which enables the detection of uniparental heterodisomy. Trio-testing, which involves testing of the proband’s biological parents, can help in the interpretation of results for de novo variants in deep intronic and other non-coding regions.

Finally, when discussing the diagnostic effectiveness of WGS, genetic mosaicism must be taken into account. The issue lies in the fact that WGS analysis is most commonly performed on a peripheral blood sample, or one of the other alternatives if necessary. The precision of WGS in this clinical scenario was analyzed by King DA et al. in a paper published in 2017 [114]. The authors examined a large group of patients with undiagnosed developmental disorders. In 73% of mosaic events, there was a difference in results between the peripheral blood and saliva samples, suggesting that the blood sample alone would miss a considerable fraction of chromosomal abnormalities.

These clinical examples highlight the distance that still needs to be covered in terms of research before WGS can be fully utilized as a clinical tool. While it produces considerably large amounts of data, it still needs to be approached with caution. The greatest issues with using this technology incautiously boil down to misinterpretation of the detected abnormalities or overlooking undetected ones.

## 9. Fourth-Generation Technologies and Future Directions

With the introduction of next-generation sequencing, sequencing yield increased along with a decrease in sequencing cost. Most of these genomes were presented in small pieces. Consequently, the gene annotation in these genomes is either inadequate or nonexistent altogether. As a result, long-read sequencing was introduced, and one of the primary products on the market is nanopore sequencing by Oxford Nanopore Technologies (ONT), which has a very low cost [115,116,117].

Nanopore sequencing technology has the potential to make nucleic acid sequencing accessible and feasible for everyone. An obstacle stands in the way: interpreting nanopore sequences requires high bioinformatics skills. However, as interpretation technologies advance and biologists expand their bioinformatics knowledge, the potential of nanopore sequencing is sure to keep evolving [118,119].

Single-cell genomics and spatial transcriptomics are important tools revolutionizing genome sequencing. These tools assist in measuring gene activity, mapping the activity, and monitoring the resultant molecular phenotypes. Single-cell genomics is the study of cellular uniqueness and utilizes omics techniques such as single-cell RNA sequencing (scRNA-seq) and single-cell DNA sequencing (scDNA-seq), which allow for the analysis of genetic variants and gene expression patterns at the single-cell level [120]. Spatial transcriptomics features other techniques, including in situ hybridization, digital optical barcoding, conventional immunofluorescence methods, and next-generation sequencing [121]. Single-cell genomics possesses the potential to expand the current knowledge of disease pathogenesis, opening the door for improved personalized medicine and targeted therapeutic interventions [120,121]. Similarly, spatially resolved transcriptomics has the potential to supply a thorough understanding of the molecular architecture of tissues, providing novel insights into organ growth, function, and disease mechanisms [122].

Multi-omics integration is the practice of integrating and analyzing multiple omics datasets in a clear and logical manner to address the obstacles of organizing and managing large amounts of data without errors [123]. Omics has opened the door for advanced data analysis, resulting in exciting opportunities, breakthroughs, and challenges for both statisticians and biologists. However, in order to achieve quality results from multi-omics, experiments must be carefully designed, data must be diligently collected, and findings must be FAIR (findable, accessible, interoperable, and reusable). The goal of multi-omics integration is to incorporate that into precision health: an individualistic approach that integrates data from medical history, omics, environment, lifestyle, and other factors. Precision health involves generating the data and modeling them, and multi-omic integration will provide greater insight, resulting in more accuracy in precision health [124,125,126].

## 10. Clinical Experiences with NGS Diagnostics

The clinical utility of WGS lies in its ability to detect genetic variants in coding regions, non-coding regions, as well as structurally complex variants such as deep intronic variants. In our clinical practice, we have had multiple cases where next-generation sequencing (NGS) has proven to be an essential diagnostic tool. By integrating multi-omics data, including genomics, metabolomics, and proteomics, we have significantly enhanced our diagnostic capabilities.

For instance, in one case involving a patient with severe and deteriorating neurological symptoms, the combination of WGS and metabolic profile allowed us to identify a novel pathogenic variant in a non-coding region of the genome, shedding light on the molecular basis of the condition [127]. Additionally, in cases of undiagnosed genetic syndromes, the integration of genomics data provided a comprehensive view of the underlying molecular mechanisms, aiding in the accurate diagnosis and subsequent management of these conditions. WGS presently facilitates precise diagnostics of rare diseases in cases such as uniparental isodisomy among children with Prader–Willi and Angelman syndromes, de novo deep intronic variants, and repeat expansions in non-coding regions among individuals affected by diseases such as myotonic dystrophies. Moreover, our experience extends to cases where traditional diagnostic approaches failed to provide conclusive results [128,129]. The synergy of genomics, metabolomics, and proteomics has been instrumental in uncovering elusive genetic mutations and intricate molecular signatures that would have otherwise gone undetected.

In summary, the integration of multi-omics data, facilitated by advanced sequencing technologies like WGS and NGS, has been a transformative approach in our clinical practice. It has enabled us to unravel complex genetic landscapes, leading to more accurate and personalized diagnoses in diverse clinical scenarios.

## 11. Conclusions

The application of WGS holds significant potential in the field of molecular medicine, shaping the future of genetic disease diagnosis. The rapid advancement in genome sequencing technology has enabled increasingly rapid and high-quality genome analysis, characterized by high precision and diminishing costs. The incorporation of WGS into routine clinical practice presents novel opportunities for personalized medicine and improved patient health outcomes, including proactive measures to prevent the development of multifactorial diseases. Looking ahead, WGS is expected to become a standard diagnostic tool in pediatrics, facilitating precise and personalized care for children with monogenic and multifactorial diseases. The integration of WGS into clinical practice represents a significant paradigm shift, offering hope for improved outcomes for individuals grappling with rare diseases. This powerful technology not only enhances diagnostic accuracy but also opens new avenues for personalized treatments, ultimately paving the way for a brighter future for patients around the world.

## Data Availability

No new data were created in the creation of this article.
